# Cell-Wide DNA De-Methylation and Re-Methylation of Purkinje Neurons in the Developing Cerebellum

**DOI:** 10.1371/journal.pone.0162063

**Published:** 2016-09-01

**Authors:** Feng C. Zhou, Marisol Resendiz, Chiao-Ling Lo, Yuanyuan Chen

**Affiliations:** 1 Department of Anatomy & Cell Biology, Indiana University School of Medicine, Indianapolis, Indiana, 46202, United States of America; 2 Stark Neuroscience Research Institute, Indiana University School of Medicine, Indianapolis, Indiana, 46202, United States of America; University of Kentucky, UNITED STATES

## Abstract

Global DNA de-methylation is thought to occur only during pre-implantation and gametogenesis in mammals. Scalable, cell-wide de-methylation has not been demonstrated beyond totipotent stages. Here, we observed a large scale de-methylation and subsequent re-methylation (CDR) (including 5-methylcytosine (5mC) and 5-hydroxylmethylcytosine (5hmC)) in post-mitotic cerebellar Purkinje cells (PC) through the course of normal development. Through single cell immuno-identification and cell-specific quantitative methylation assays, we demonstrate that the CDR event is an intrinsically scheduled program, occurring in nearly every PC. Meanwhile, cerebellar granule cells and basket interneurons adopt their own DNA methylation program, independent of PCs. DNA de-methylation was further demonstrated at the gene level, on genes pertinent to PC development. The PC, being one of the largest neurons in the brain, may showcase an amplified epigenetic cycle which may mediate stage transformation including cell cycle arrest, vast axonal-dendritic growth, and synaptogenesis at the onset of neuronal specificity. This discovery is a key step toward better understanding the breadth and role of DNA methylation and de-methylation during neural ontology.

## Introduction

Cytosine methylation is directly involved in modulating transcriptional activity and other genome functions[1]. Once established, DNA methylation is believed to be a relatively stable epigenetic modification as global, cell-wide alterations in DNA methylation have only been observed during two early stages of life: pre-implantation development and gametogenesis [2–8]. The first wave of cell-wide de-methylation occurs asynchronously between the maternal and paternal genomes in pronuclear staged embryos, with the paternal genome becoming de-methylated rapidly following fertilization, and the maternal genome undergoing sequential replication-mediated de-methylation through the blastocyst stage [9]. Due to the lack of *de novo* (DNMT3) and maintenance (DNMT1) methyltransferases in pre-implantation embryos, maternal and paternal chromatids continue to undergo replication-dependent loss (i.e. passive de-methylation) of both 5-methylcytosine (5mC) and 5-hydroxymethylcytosine (5hmC) as embryos progress through the 8-cell stage [10]. By the blastocyst stage, most of the genome is devoid of 5mC and 5hmC, except for specific imprinting control elements, genes undergoing X-inactivation, and repetitive DNA elements (e.g. transposons). The second wave of cell-wide de-methylation occurs in primordial germ cells (PGCs), and begins at embryonic day (E) 8.5 in mice and continues through E12.5 as cells migrate to the gonadal ridge [2, 5–8, 11]. High-resolution methylome studies of murine PGCs indicate that some genomic elements escape de-methylation, as 6–10% of CpGs remain methylated in female PGCs, while 16–20% escape reprogramming in male PGCs [8]. In addition, a large percentage (25% -30%) of the LTR-ERV1 and LTR-ERVK transposons, including intracisternal alpha particle (IAP) elements, resist de-methylation [8, 11]. These studies demonstrate that ‘global de-methylation’ is not 100% penetrant, and that de-methylation timing is not uniform across the genome. Epigenetic reprogramming is completed when de-methylated strands further undergo *de novo* methylation by the enzyme DNMT3a and 3b during male and female gametogenesis [5].

The occurrence and purpose of these cellular de-methylation and re-methylation (CDR) events are enigmatic at best. Until recently, it was believed that CDRs only occur at germinal stages of development, though the case for epigenetic reprogramming of mature cells has begun to gain traction [12]. This is fundamentally important, as the methylation program is a potential upstream mechanism for cell fate determination as well as cellular differentiation and gene regulation beyond cellular maturity. In that vein, a few investigations have reported replication-independent DNA de-methylation in post-mitotic blood cells, often associated with terminal stages of differentiation [13]. DNA methylation changes observed in mature neurons thus far have been largely attributed to the fluctuating burdens of neuronal activity, such as those occurring in learning and memory formation [14–16]. One study, however, has indicated that there is a naturally occurring, wide-scale genomic decrease of methylation in the aging human prefrontal cortex [17]. Additionally, indirect post-mitotic neuronal de-methylation has been suggested by intrinsic age (stage)-dependent acquisition of 5hmC in the cerebellum and hippocampus [18, 19].

We have previously demonstrated that DNA methylation during early prenatal neural development is not a random event, but rather progresses in an orderly spatiotemporal program that coincides with cellular restriction and differentiation in the neural tube at the neurulation stage in mice [20, 21]. The initiation of neural progenitor cell differentiation was highlighted by a dramatic escalation of 5mC, and particularly 5hmC, throughout the neuroepithelial layer [22, 23]. Here we further present evidence of an extensive, cell-wide DNA methylation reprogramming event occurring during non-proliferative neuronal differentiation and as a normal program during late-stage differentiation of the cerebellar Purkinje cell in mice. While others have described unilateral post-mitotic de-methylation, this is the first report of a CDR that occurs outside of totipotent or germ cells, and is characterized by a marked sequential reduction and acquisition of 5mC and 5hmC. It is proposed herein as a component of the normal transition of post-mitotic neurons at the peak of synaptogenesis which occurs in a cell-type specific manner at the active brain wiring stage. This neuronal CDR, at varying levels, may serve as a mechanism underlying scheduled temporal gene expression for neuronal-type specific differentiation, migration, axonal-dendritic growth and synaptogenesis.

Due to the sizeable nucleus, known cytological migration path, clear position in the Purkinje cell layer (PCL), and characteristic and protracted temporal development and synaptogenesis, the Purkinje cells provide a magnified view for elucidating the features of cell specific epigenetic programs. The PCs, together with distinct cerebellar granule cells (and a small fraction of interneurons), clearly demonstrated a cell specific epigenetic evolution occurring within the stratified layers and cyto-architecturally repetitive 3D arrangement of the developing mouse cerebellum. In light of this novel observation, we adopted additional molecular and single cell population analyses to demonstrate the novel and unique epigenetic landscapes of maturing cerebellar neurons.

## Materials and Methods

### Animals and Immunohistochemistry

C57BL/6J mice (Jackson Laboratories) were used in this study under protocol MD/R 11037. The study has been approved by the Indiana University School of Medicine Animal Care and Use Committee. Animals were group-housed in the IUSM Laboratory Animal Resource Center under a reverse light/dark cycle (10:00–22:00) and standard laboratory chow diet. A two-hour mating paradigm (2 females to 1 male) was used to obtain pups. Pregnancies could thus be tracked to the nearest two-hour interval of conception. The day of birth is denoted as postnatal day (P) 0. In this study, six P7, four P14, six P21, four P28, and four P45 mice were selected from at least four different litters, anesthetized by CO2 inhalation to alleviate pain (sedation confirmed by tail-pinch test) and transcardially perfused with saline, followed by 4% paraformaldehyde. Finally, cervical dislocation was performed as a secondary means of euthanasia. Adult dams were consequently anesthetized by CO2 inhalation (sedation confirmed by tail-pinch test) followed by cervical dislocation to ensure euthanasia. Pup brains were dissected and post-fixed for a minimum of 48 hours. Brains were subsequently embedded in 10% gelatin blocks and sectioned by free-floating vibratome (Leica VIT100S) at 40um, coronally.

Immunohistochemistry was used for the identification of methylation marks. Antibodies used in this study were: 5mC (mouse monoclonal, 1:2500, Eurogenetec, Seraing, Belgium), 5hmC, 5caC, and 5fC (rabbit polyclonal, 1:3000, 1:2000, and 1:2000 respectively, Active Motif, Carlsbad, CA), MBD1 (rabbit polyclonal, 1:200, Santa Cruz Biotechnology, Santa Cruz, CA), MeCP2 (rabbit monoclonal, 1:1000, Cell Signaling Technology, Danvas, MA), TET1 (rabbit polyclonal, 1: 500, GeneTex, Irvine, CA) and DNMT1 (1:1000 rabbit polyclonal, Abcam Cambridge, MA*)*. To address issues including staining penetration and inherent reduction of PC immunoreactivity, we performed an immunostain using the Purkinje cell marker calbindin-D28K (1:1000, Bioss Antibodies, Woburn, MA). Biotinylated secondary antibodies (Jackson ImmunoResearch, West Grove, PA) used at 1:500 for 90 minutes after primary antibody incubation, followed by 90 minutes of incubation in biotin-streptavidin conjugated tertiary antibodies (1:500). Optical detection of immunoreactivity was accomplished by 3,3’-Diaminobenzidine (Sigma-Aldrich, St. Louis, MO) activated by 3% hydrogen peroxide. Mounted sections were dried, counterstained by Methyl-green and preserved by serial alcohol dehydration and coversliped with Permount (Fisher Scientific, Waltham, MA). Both Bright-field and Olympic Confocal Microscopies were used to visualize the immunostaining.

In some cases, Nissl stains such as Methyl Green have been known to be harder to stain in mature Purkinje cells compared to granule cells of the IGL[24]. Here the existence of Methyl Green can be demonstrated by prolonged staining as demonstrated, however, Purkinje cells are clearly demarcated by basket cells, which are known to surround the cell bodies of Purkinje cells [25]. Additionally, PC cell bodies were also detectable in the absence of nuclear immunoreactivity through the presence of a light Nissl stain (amplified under Brightfield microscopy) and the neuronal perikarya.

### Confocal Microscopy

Confocal fluorescent images were obtained with an Olympus FV1000-MPE Confocal Microscope mounted on an Olympus IX81 inverted microscope stand with a 60x water-immersed objective. Sequential excitation at 488 nm and 559 nm was provided by argon and diode lasers, respectively. Emission was collected by spectral detectors in channels one and two with user-specified min and max wavelengths. Z-stack images were collected over a thickness of 4.5 μm in 0.3 μm step intervals. After sequential excitation, green and red fluorescent images of the same cell were saved and analyzed by Olympus Fluoview FV10-ASW software. The term co-localization refers to the overlap of green and red fluorescence in an image pair, as measured by confocal microscopy.

### Quantification of 5mC and 5hmC using molecular enzymatic assays

To independently assess quantitative cellular methylation, 25μm coronal sections were obtained from flash-frozen cerebellum at ages P8 (N = 8) and P29 (N = 8) using a Leica Cryostat CM1900. Sections were mounted onto 2μm thick PEN membrane slides (Microdissect Gmbh, Herborn, Germany) and stained with 4X Thionin solutions. Slides were further dehydrated in series 50%, 75%, 90%, and 100% ethanol. Sections were viewed under 10X magnification using a Leica laser microdissection microscope (Leica CTR6500). The cerebellar layers EGL, IGL and PCL were morphologically discernible by Thionin stain and dissected by laser into a flat cap 0.65mL Eppendorf tube containing 65μL of DNA extraction buffer (Arcturus Picopure DNA Extraction kit, Applied Biosciences). DNA was processed according to manufacturer’s instructions. DNA was next purified using DNA Clean and Concentrator (Zymo, Irving, CA) according to manufacturer’s instructions.

100ng and 200ng of purified cellular gDNA was used for MethylFlash^™^ Methylated DNA Quantification Kit and MethylFlash^™^ Hydroxymethylated DNA Quantification Kit respectively, according to manufacturer’s instruction (Epigentek, Farmingdale, NY). Absolute quantification was performed using a five-point standard curve and further transformed to represent the percentage of methylated DNA (5mC or 5hmC) in the total DNA.

### Quantitative Gene Specific DNA methylation analysis

2 μg of genomic DNA were extracted from cerebellum samples and digested. There were four treatment groups for each sample: uncut, methyl-sensitive enzyme (MSRE; *Hha* I or *Hpa* II; New England Biolabs, Ipswich, MA), methyl-dependent enzyme (*Mcr*BC; New England Biolabs, Ipswich, MA), and double digest with a methyl-sensitive and a methyl-dependent enzyme. Each digest was conducted with 10 U enzyme and incubated for 12 hours at 37°C. In the double digest sample, the first digest was using the methyl-sensitive enzyme, and the enzyme was heat inactivated by incubating at 65°C for 20 minutes. Prior to the second digestion, we purified the singly-digested DNA by phenol: chloroform extraction, and then precipitated the samples with 0.2 volumes of 1.5 M ammonium acetate and 2 volumes of 100% ethanol and re-suspended them in 1 X T_10_E_1_ buffer. Prior to amplification, all DNA were purified by phenol: chloroform extraction followed by ethanol precipitation and quantified by Nanodrop ND-1000 Spectrophotometer (NanoDrop Products, Wilmington, DE). We used 40ng of DNA from each digestion for quantitative PCR reaction with iTaq Universal SYBR Green mix (BioRad, Hercules, CA) and locus-specific primers using an iCycler PCR machine (BioRad, Hercules, CA). We used the default setting to obtain the cycle threshold (Ct) values, and normalized the digested samples to the uncut samples to calculate the delta Ct value. DNA methylation levels were determined by the average MSRE delta Ct value based on the calculation of 100 x 2^-ΔCt^. Three or four biological replicates for both P7 and P29 animals were used for this analysis.

### qPCR for gene expression

We added 10 volumes of RNA Stat-60 (Tel-Test, Inc., Friendwood, TX) to the snap-frozen P7 or P29 cerebellum, homogenized and incubated the sample at room temperature for 5 minutes. To purify the RNA, we extracted the samples for 5 minutes with 0.2 ml chloroform/ ml RNA Stat, and centrifuged the samples for 15 minutes at 12,000 RPM (max) at 4°C. We transferred the aqueous layer to a new tube and precipitated the RNA for 10 minutes at room temperature with 0.5 ml isopropanol/ ml RNA Stat and then centrifuged the sample for 10 minutes at 12,000 RPM (max) at 4°C. The pellet were washed with 75% ethanol, vortexed, and centrifuged for 5 minutes at maximum speed at 4°C. We dried the pellet and dissolved it in 300 μl DEPC H_2_O. RNA concentration was measured by NanoDrop ND-1000 Spectrophotometer (NanoDrop Products, Wilmington, DE). We performed DNase treatment using the TURBO DNA-free kit (Life Technologies, Grand Island, NY) to one μg of total RNA extracted from each sample by following the manufacture’s instruction. DNase treated RNA was then converted into cDNA using iScript cDNA synthesis kit (Bio-Rad, Hercules, CA). *Quantitative RT-PCR* was performed using 50 ng of cDNA as a template for qRT-PCR in combination with TaqMan^®^ Gene Expression Master Mix (Life Technologies, Grand Island, NY) and TaqMan Gene-specific probes (Life Technologies, Grand Island, NY) listed below on a StepOnePlus^™^ Real-Time PCR System (Life Technologies, Grand Island, NY). We assayed four biological replicates for P7 and P29. Cycling reactions were performed in duplicate. The relative expression of each gene was calculated based on the ΔΔCt value, where the results were normalized to the average Ct value of *Gapdh*.

### Statistical analysis

A randomized design with a one–way arrangement of the treatments, "Age (P7 & P29)" data, was analyzed through the Generalized Linear Model procedure on SAS and comparisons between treatments were done through the Least Square Means procedure.

All quantitative data herein were represented as the mean and standard error of the mean (SEM). Statistical analysis was performed between P8 and P29 DNA using a one tailed student t-test assuming unequal variance (GraphPad, Prism 6.0). The threshold for statistical significance was p<0.05.

## Results

### The Prominent Purkinje Cells

#### The de-methylation history of Purkinje cells

**Fig 1:**
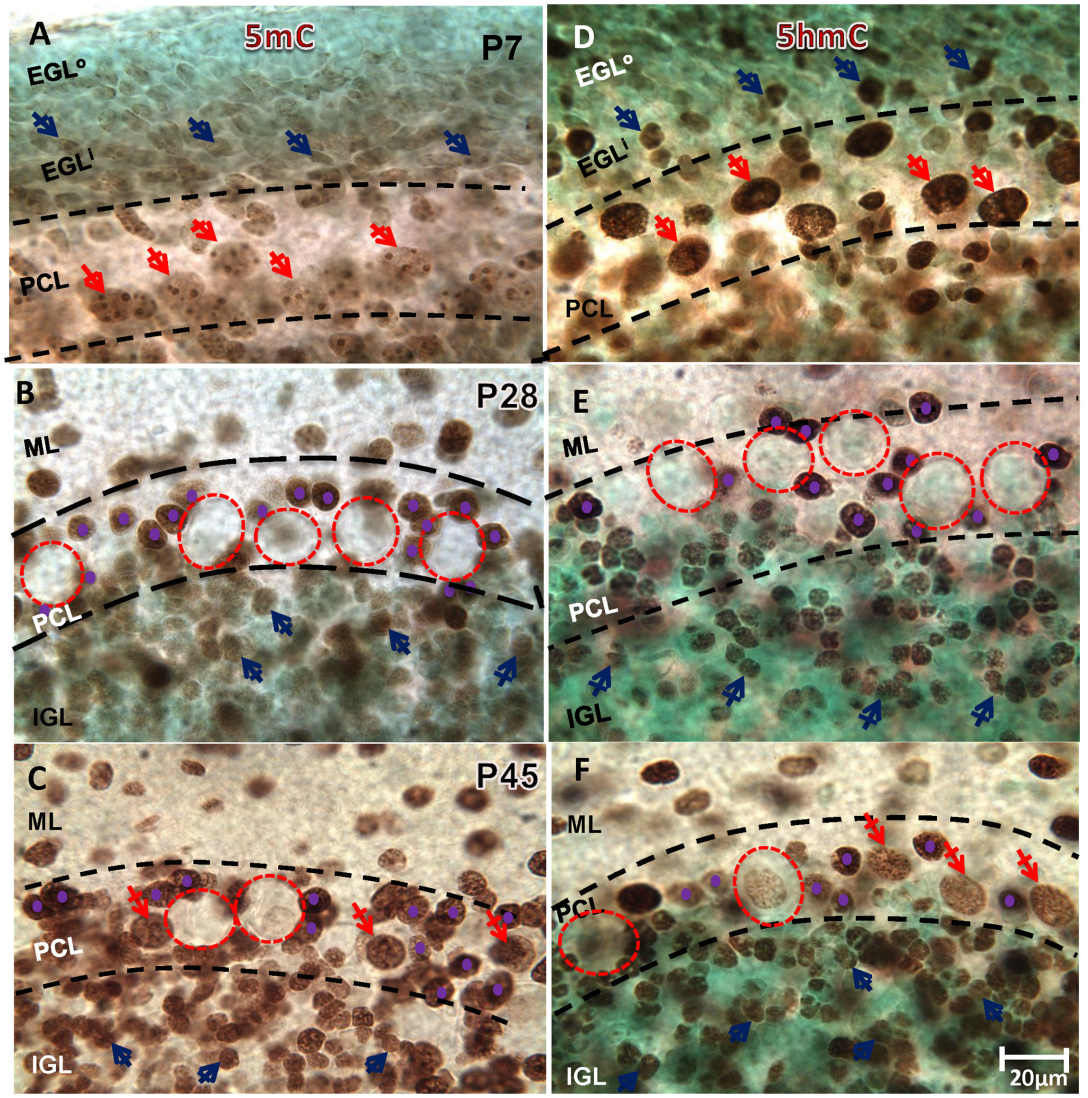
Cell-Specific DNA Methylation profiles of the post-natal cerebellum. (**A**) 5mC-immunostaining (im) is intensively present in the nucleus (size >12μm) of postmitotic Purkinje Cells (PC) at P7 (red, crossed arrows) at PC layer (PCL). 5mC is also distinctively present only in the non-dividing granule cells of the inner portion of the External Granule Layer (EGLi, blue crossed arrows) but not outer portion of EGL (EGLo). (**B)** By P28, the waning of 5mC-im was evident in PCs (red, dashed circles; ~20μm diameter). Basket cells surrounding the PCs (purple dots, <8μm) were intensively immunostained by 5mC (as well as all other interneurons). Mature granule cells inhabiting the Inner Granule Layer (IGL) retained the acquired 5mc-im throughout the remainder of the time-course. **(C)** By P45 re-methylation of PCs occurred as the 5mC-im returned to some but not to all de-methylated PCs (red, crossed arrows denote re-methylated PCs). (**D**) At P7, 5hmC immunostaining (im) is intensively present in PCs (red, crossed arrows), though distributed distinctly from 5mC. Some granule cells of the inner EGL express 5hmC-im though not at the upper surface of the EGL, where granule cell progenitors reside. **(E)** At P28, a clear de-methylation of 5hmC occurs in the PCs (red, dashed circles) as occurs with 5mC. Granule cells which have migrated to the IGL continue to acquire 5hmC (blue, crossed arrows) as do the emerging basket interneurons surrounding the perimeter of PCs (purple dots). (**F**) At P45, re-methylation of 5hmC occurs in line with 5mC re-methylation at PCs (red, crossed arrows denote re-methylated PCs). Interneurons and granule cells appear to refrain from de-methylation throughout their developmental time-course. Scale bars: **A-F** = 20μm; Methyl Green Nissl counterstain. Dashed red circles depict the approximate boundaries of the PC cell body. Dashed black lines depict approximate boundaries of the PCL.

During early neurulation, neuroepithelial 5mC-immunostaining (im) increased first and were followed by ~ 1 day by 5hmC-im between E15 and E17 in the nuclei of differentiating PCs, after which both of the methylation markers were progressively increased (data not shown). This corresponded to the stage when Purkinje cells migrate in transit to the beginning monolayer arrangement of the Purkinje Cell Layer (PCL). 5mC-im and 5hmC-im were markedly increased during this migration/arrangement, peaking between postnatal day 4 (P4) and P7 (Fig 1A and 1D). During the following weeks, virtually all PCs (while forming apical dendrites) underwent a decrease of 5mC-im, followed by a reduction in 5hmC-im. Between P14 and P28 specifically, 5mC-im (Fig 1B) and 5hmC-im (Fig 1E) were drastically decreased in PCs in a noticeable gradient (from the deep folia to the surface of the cerebellum) which coincided with the known maturation gradient of PCs [26, 27]. This phenomenon was observed consistently throughout the rostro-caudal axis of the cerebellum. Overall, this time period parallels the characteristic, robust outgrowth of the extensive PC dendritic arbors and parallel fiber synaptogenesis [28, 29]. By P28, at the culmination of PC functional maturity [30] and the onset of PC stabilization [29], most of the PCs appeared devoid of both 5mC-im (Fig 1B) and 5hmC-im (Fig 1E) marks. In contrast to PC de-methylation, basket cells (which surround Purkinje neurons) and molecular layer (ML) interneurons, along with granule cells, demonstrated an increase in nuclear 5mC-im and 5hmC-im expression during their respective developmental transitions (Fig 1B and 1E). By P45, as synaptogenesis was mostly completed, PCs became re-methylated gradually with 5mC **(**Fig 1C) followed by 5hmC (Fig 1F), though they never reached levels comparable to peak methylation. Overall, these results indicated at least two distinct and developmentally relevant DNA methylation programs highlighting a cell-wide, CDR event occurring on post-mitotic neurons.

**Fig 2:**
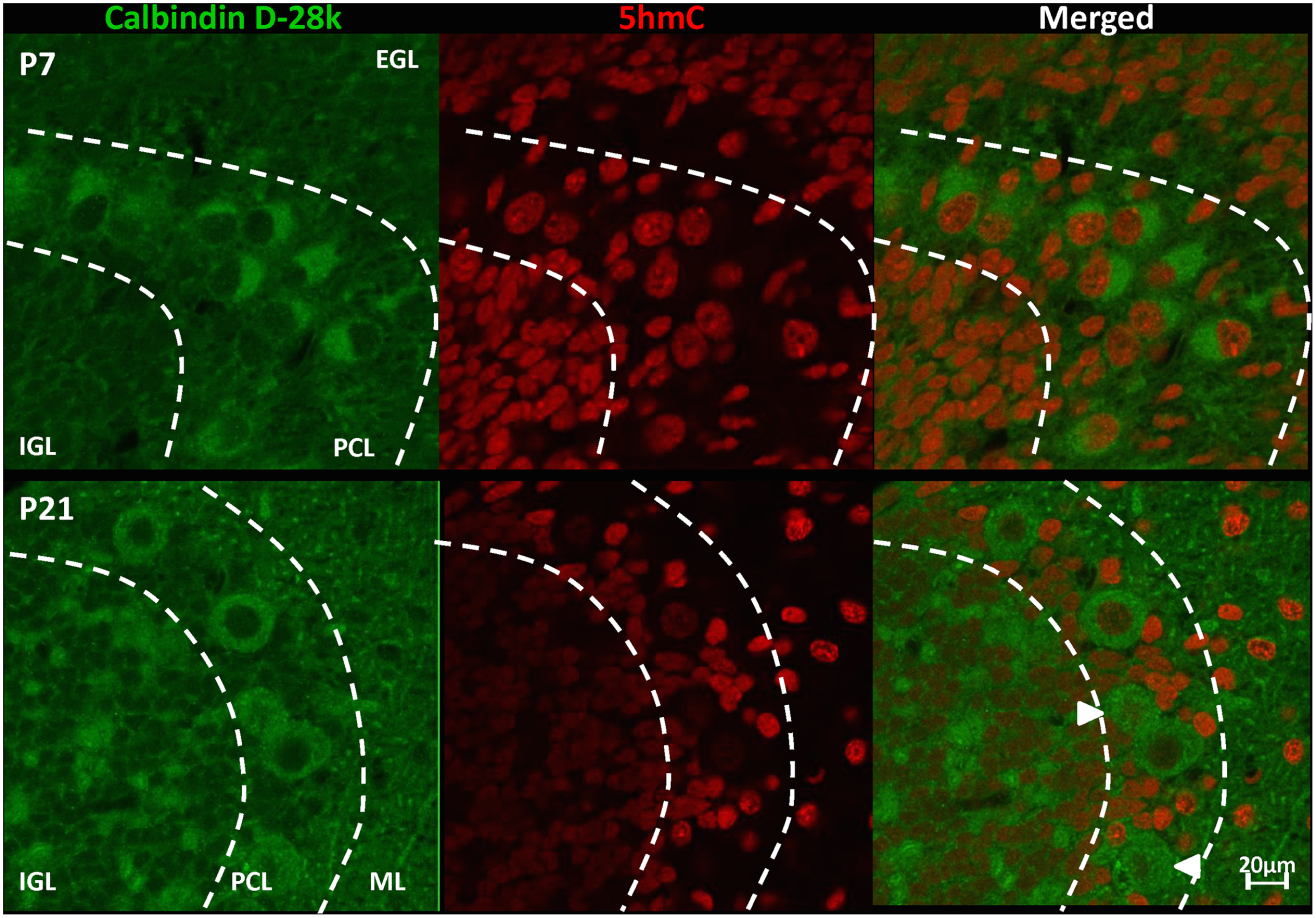
Calbindin D-28k and DNA methylation co-labeling in the developing cerebellum. The calcium-binding protein Calbindin D-28k (green) is a characteristic Purkinje cell protein and appears markedly immunoreactive at P7 in the PCL. At P7, 5hmC (red) is also abundant in PC nuclei and granule cells of the inner EGL and IGL. At P21, even while an abundance of DNA methylation markers undergo dramatic loss of immunoreactivity, Purkinje cells retain calbindin expression. In contrast, interneurons emerging in the ML (as well as basket cells surrounding the large Purkinje bodies) abundantly express 5hmC at P21. EGL: external granule layer, IGL: internal granule cell layer, PCL: Purkinje cell layer, ML: molecular layer. Dashed borders represent the approximate cytological borders of the PCL. Scale bar = 20μm.

To ascertain that the penetrance and immunoreactivity of the PCs was bona fide during de-methylation, we performed a double-immunolabeling for the calcium-binding protein and PC marker calbindin-D28K and 5hmC. At P7, an abundance of calbindin-D28K-im was observed in the PC cell body which was simultaneously expressed alongside nuclear 5hmC (Fig 2C). At P21, during observed de-methylation of the PCs, calbindin-D28K-im is retained in the large PC cell body while nuclear DNA methylation, marked by 5hmC, is dramatically decreased (Fig 2F). Interestingly, 5hmC during PC de-methylation is abundantly expressed in burgeoning interneurons of the molecular layer (ML) as well as basket cells (interneurons) surrounding the large PC cell bodies in the PCL (Fig 2E). 5hmC and calbindin co-labeling was evaluated as a representative pattern of other, relevant DNA methylation markers since it was expressed parallel to 5mC (Fig 1), DNMT1, and Tet1.

#### Cell-specific molecular DNA methylation analysis

**Fig 3:**
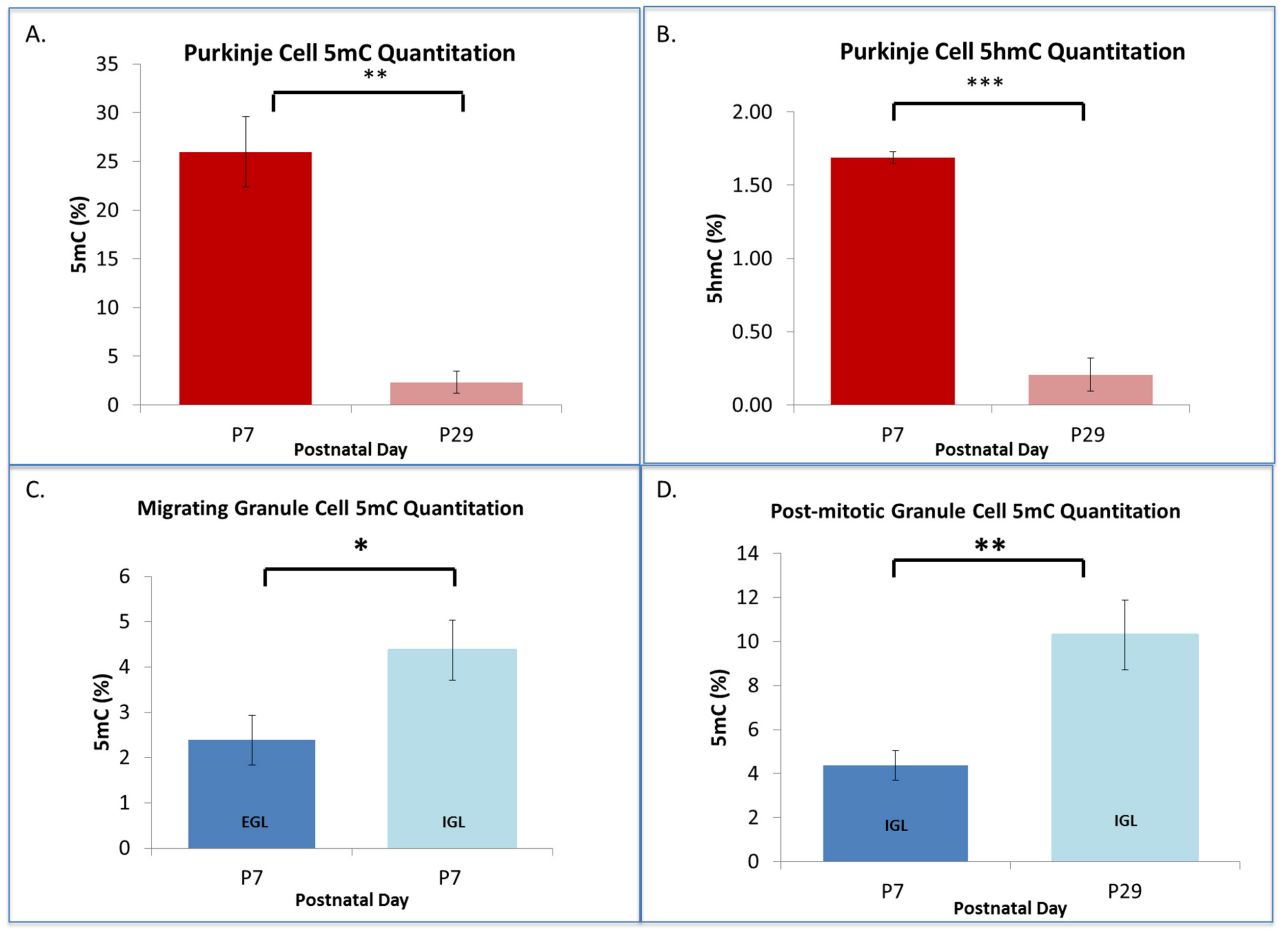
Quantitative Detection of Cell-Specific, Developmental DNA Methylation in the Cerebellum. Purified gDNA obtained from laser micro-dissected Purkinje and Granule cells was quantitatively analyzed for 5-methylcytosine and 5-hydroxymethylcytosine content (%) via antibody-based colorimetric assay. (**A-B**) Purkinje cells undergo remarkable loss of both 5mC and 5hmC between the first and fourth post-natal weeks, coincident with the Purkinje cell morphological and transcriptional transformation. (**C-D**) Granule cells of the external granule surface, as they undergo radial migration into the internal granule layer and become post-mitotic, acquire 5mC as indicated by earlier immunohistochemical analysis (Fig 1). Further, granule cells of the internal granule layer (IGL) continue to acquire methylation between the first and fourth postnatal week as granule cells settle into their mature state. All values represented as mean ± SEM (**A**.) **P-value = 0.0078; (**B**.) ***P-value = 0.0001; N = 4 per age. (**C**.) *P-value = 0.0186; (**D**.)** P-value = 0.0036; N = 8 per age.

To independently validate the observations of the cell-specific DNA methylation programs discussed above, we further quantified DNA methylation using the MethylFlash^™^ Methylated DNA Quantification assay (Epigentek) for 5mC and 5hmC paired with laser-capture microdissection (LC-MD) as indicated in the Methods. The DNA methylation of LC-MD dissected PC populations underwent a 23.64% decline in genomic 5mC (Fig 3A, p<0.01), and a great drop (8-fold) of 5hmC from P7 to P29 (Fig 3B, p<0.0001). These results are consistent with the PC immunostaining patterns observed in Fig 1 and confirmed the DNA methylation reprogramming in the post-mitotic PCs.

#### Chromatin remodeling in the cerebellar nuclei

**Fig 4:**
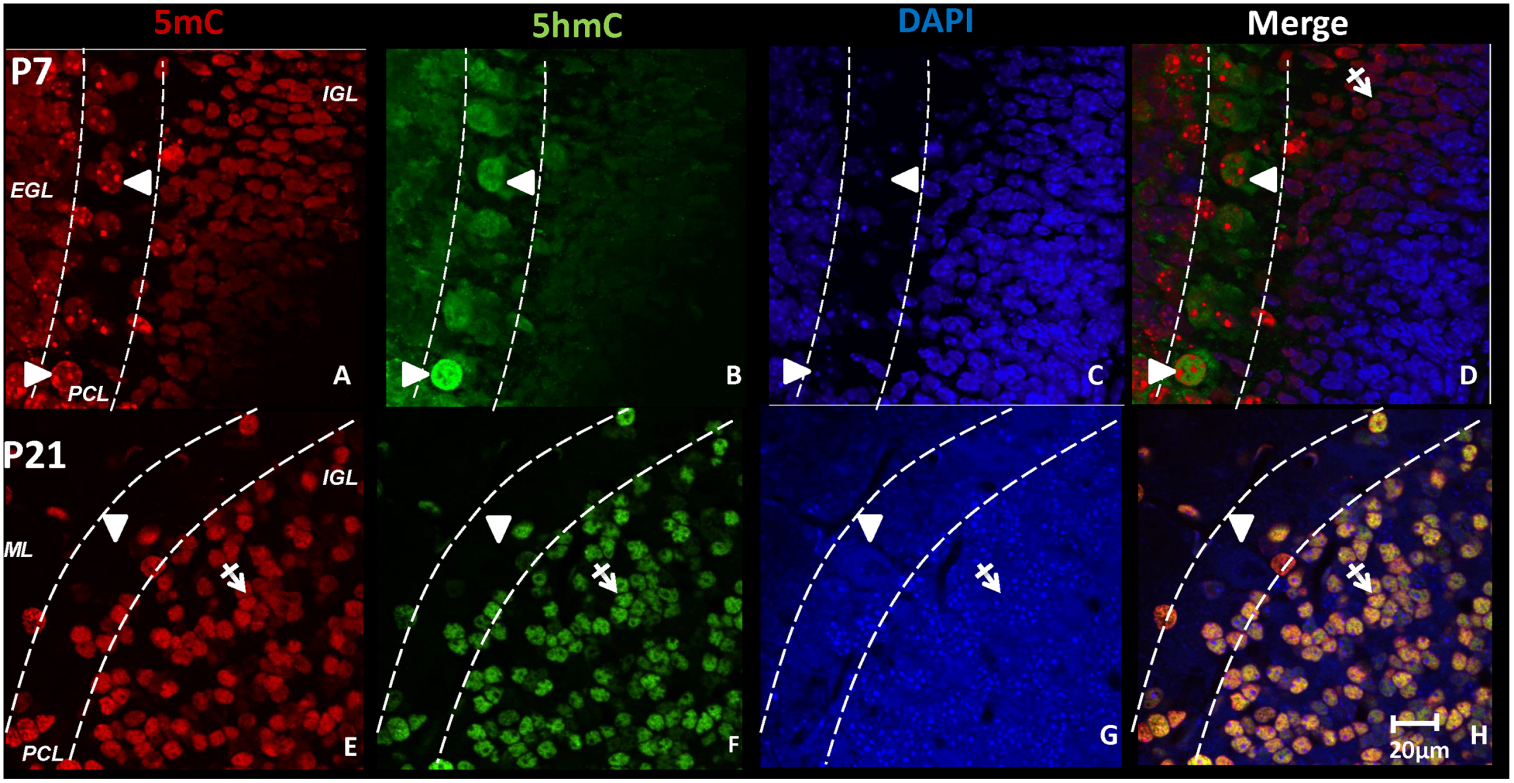
DNA methylation dynamics and chromatic remodeling of Purkinje cells during differentiation in the cerebellum. While distributed throughout the nucleus at the embryonic stage (not shown), at P7, major 5mC-immunostaining (im) (red) is packed into large punctates in the DAPI dense (blue), heterochromatic regions of the PCs located in the Purkinje Cell Layer (PCL) (**A, white arrows**). (B) 5hmC (green) was detected primarily in the euchromatic DAPI sparse regions (**B, white arrows**). At P7, as the granule cells in the external granular layer (EGL) migrate into the internal granular layer (IGL), 5mC appears to precede 5hmC expression, as no overlap was observed at P7 (**D, crossed arrows**). De-methylation of 5mC and 5hmC in PCs progresses through P14 and peaks between P21 and P28. Notice the loss of 5mC and 5hmC in most of the PCs in the PCL (**E, F white arrows**). In contrast, DNA methylation in granule cells is independent of the PC program. At P21, when migration from the EGL has ceased and cells have permanently settled in the IGL, there is significant overlap between 5mC and 5hmC as denoted by yellow fluorescence (**H, crossed arrows**). Furthermore, by P21 the characteristic punctate staining of 5mC observed during the first postnatal week has been replaced by a more homogenous distribution in the matured, rounded nuclei of the granule cells. ML: molecular layer. Scale bar: **A-H** = 20μm.

Chromatin remodeling was also evident in parallel with the DNA methylation transition occurring in cerebellar PCs. Under confocal microscopy, we observed that evolving from an early (E14-P4) homogeneous distribution, 5mC-im and 5hmC-im become chromatically mutually exclusive in their distribution during the first postnatal week. 5mC-im appears prominent in large heterochromatin aggregates as indicated by punctate staining of 4',6-diamidino-2-phenylindole (DAPI-dense regions), while 5hmC-im appears as a fine particle prevalent in a euchromatic distribution (DAPI-light regions) (Fig 4A–4D). The large punctate of the 5mC chromatic distribution is large enough to be characteristically seen at P7 under bright-field microscopy (Fig 1A). However, by P21, significant changes in DNA methylation distribution patterns culminate in the erasure of 5mC-im heterochromatic regions, followed by a large reduction of 5hmC-im euchromatin in the PCL (Fig 4E–4H). Though granule cells display a contrasting developmental acquisition of 5mC-im and 5hmC-im, they also undergo chromatic remodeling of the 5mC punctates during this time, from a heterochromatic to euchromatic distribution that largely resembles 5hmC (as demonstrated by co-labeling in the IGL at P21) (Fig 4E–4H). The observed exclusivity of the intranuclear distribution of 5mC and 5hmC supports recent reports that the two marks are differentially distributed across the genome [31, 32]. While 5mC is most commonly detected at CpG-dense regions of and associated (primarily) with transcriptionally repressive elements, 5hmC has been dominantly observed in the gene bodies and actively transcribed gene regions [23]. Chromatic exclusivity is likely a biological representation of this genomic diversity.

#### Changes of methylation and de-methylation enzymes

**Fig 5:**
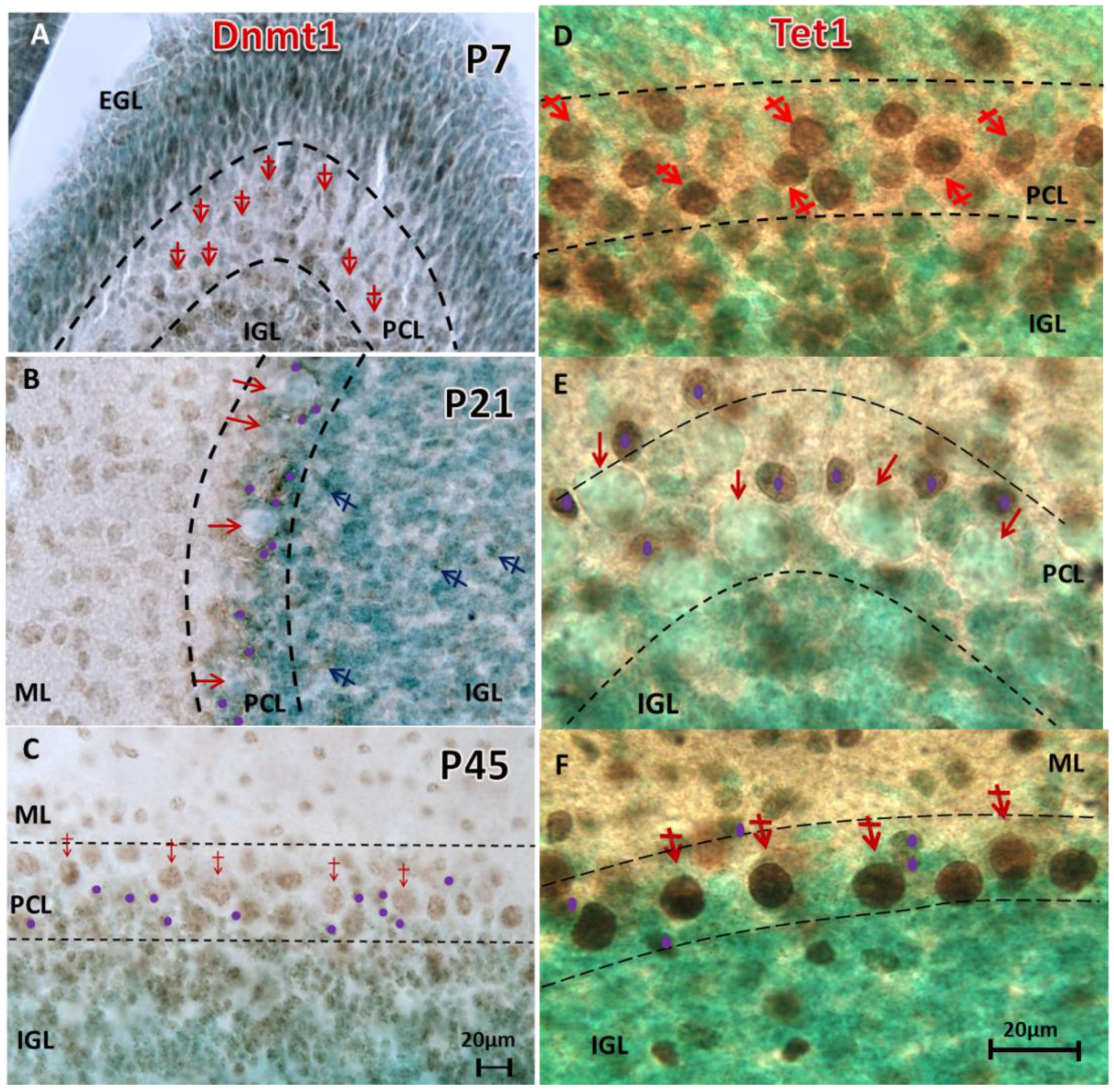
De-methylation and re-methylation are synchronized upon turnover of DNMT1 and Tet1 throughout PC maturation. The peak heterochromatic appearance of 5mC-im (Fig 1) occurs at the same time as peak Dnmt1-im (**A**, red crossed arrows). Similarly, the peak euchromatic staining of 5hmC-im (Fig 1) occurs at the same time as peak Tet1-im (**D**, red crossed-arrows) in PCs at P7. De-methylation follows progressively, as by P21 many PCs lacked DNMT1 (**B**, red arrows), and subsequently were devoid of Tet1 (**E**, red arrows). The methyl green counterstaining reveals 5hmC negative and Tet1-negative PC cell bodies (red arrows). Meanwhile, surrounding basket cells (and other interneurons) acquire Tet1 (**E**, purple dots). By P45, as re-methylation of 5mC is occurring, DNMT1-im is notably returned to the PC nuclei (**C**, red crossed-arrows). Similarly, Tet1 is observed parallel with the resumed observation of DNMT1-im expression (**F**, red crossed arrows) in PCs. PCL: Purkinje layer; EGL: external granule layer; IGL: internal granule layer. Scale bars: **A-C** = 20μm; **D-F** = 20μm.

Besides the direct observation of various DNA methylation marks, the evidence of several other markers support that neurons undergo a cell-wide de-methylation program during development. To evaluate some of these, immunostaining of the DNA methyltransferase 1 (DNMT1) and Ten Eleven Translocation Methylcytosine Dioxygenase 1 (TET1) proteins was performed. DNMT1 is responsible for conferring all of the maintenance 5mC which occurs after embryonic, *de novo* methylation. TET1 is responsible for catalyzing the hydroxylation of existing 5mC which produces the derivative 5hmC (and further, the derivatives 5-formylcytosine and 5-carboxylcytosine). At P7, DNMT1 displayed a distribution very similar to 5mC, characterized by the intranuclear punctates of euchromatin (Fig 5A). At P7, TET1 expression followed the pattern of 5hmC, denoted by heterochromatic intranuclear distribution (Fig 5D). As seen with 5mC and 5hmC, by the third postnatal week, PCs of the cerebellum were markedly devoid of DNMT1 and TET1 (Fig 5B and 5E), re-emerging again at the P45 stage to confer the subsequent re-methylation stage of CDR (Fig 5C and 5F).

**Fig 6:**
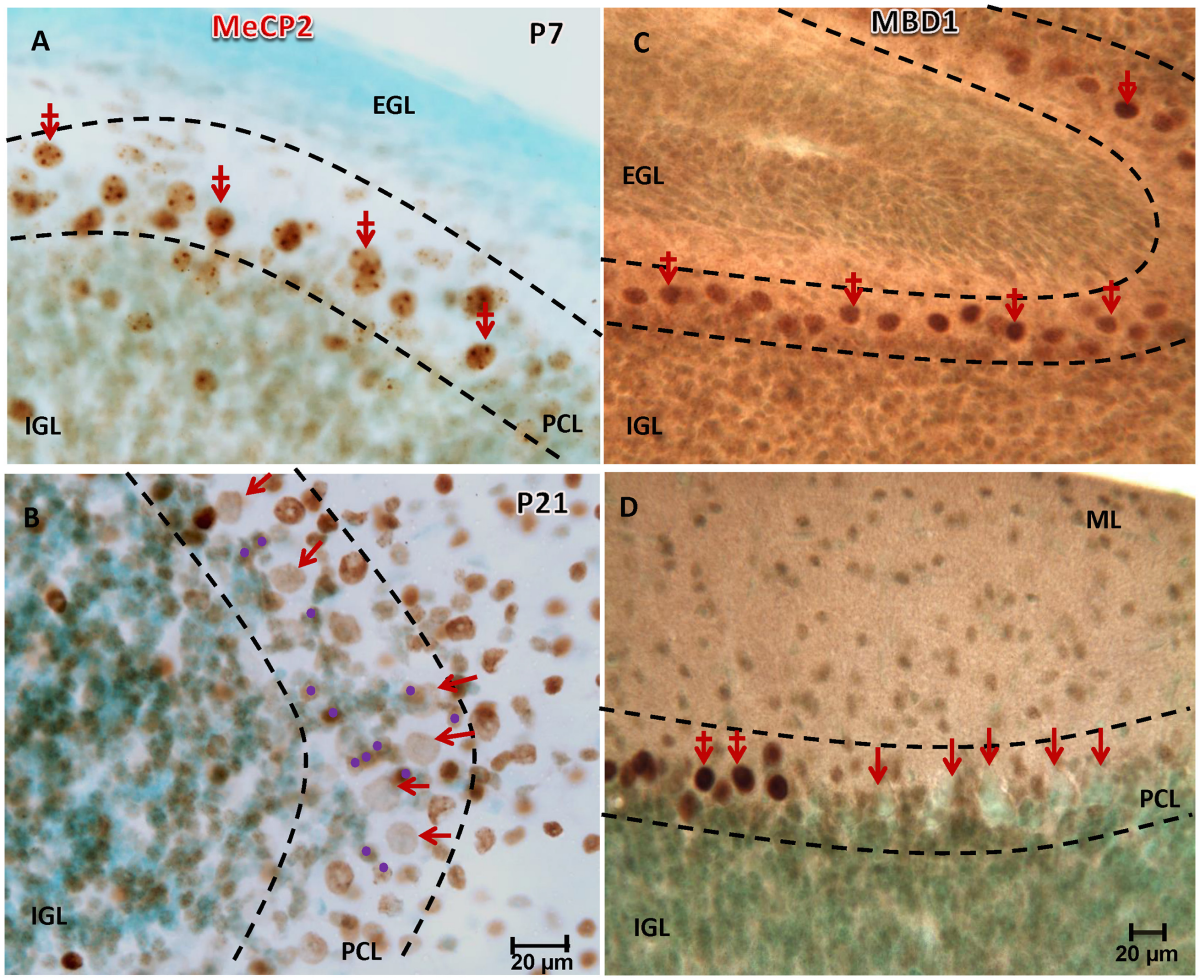
Postnatal de-methylation is supported by diminished immunoreactivity of the methyl binding proteins MeCP2 and MBD1. (**A**) Marked MeCP2 was found in the nuclei of PCs at P7 (red, crossed arrows) where its granular distribution within the nuclei is co-localized at this time point with 5mC (Fig 1). A noticeable waning of MeCP2-immunostainning in the PCs is observed by P21 (**B**, red arrows), though not to the extent observed in 5mC at the same time point. Basket interneurons (purple dots), on the other hand, acquire MeCP2 as they for a perimeter around the PCs. A similar de-methylation phenomenon is observed with MBD1-im at P21 in some but not all PCs observed (**D**, red arrows). PCs aligned in the deeper regions of the cerebellar foliae were more susceptible to this loss. IGL: internal granule cell layer, PCL: Purkinje cell layer, ML: molecular layer, Counterstaining: Nissl green. Scale bar: **A-D** = 20 μm.

Equally telling was the observation of the downstream methyl CpG binding protein 2 (MECP2), a major neuronal DNA methylation binding protein. MECP2 was present in PCs at P7 in a punctate distribution (Fig 6A), which is in agreement with the observed 5mC distribtuition. Though MECP2 in Purkinje cells does appear to undergo a noticeable amount of reduction during synaptogenesis and axonal-dendritic outgrowth, it did not match the level of reduction observed in either 5mC or 5hmC-im (Fig 6B). In addition to MECP2, the methylation binding domain protein 1 (MBD1) also depicts a loss of expression at P21, though some PCs do appear to retain this methyl binding protein as well (Fig 6D). In both cases, it appears that the protein expression loss occurs along the maturation gradient of the inner folia and cerebellar lobules. Taken together, the correlation between DNA methylation mark (5mC and 5hmC) and methyl binding protein (MBD1 and MECP2) developmental loss indicate that a major de-methylation reprogramming event occurred post-mitotically in the Purkinje neurons of the cerebellum, supported by downstream epigenetic correlates.

#### DNA-methylation at gene level

To determine if these cell-specific, cell-wide de-methylation events are reflected at the gene level, we investigated the cerebellar DNA methylation status of *Grid2*, *Cacna1g*, *Itpr1*, *Ppp1r17*, *Syt2* and *Rgs8*, of which turn-over is PC specific at P7 and P29. These genes are expressed predominantly in Purkinje cells at synaptogenesis and were chosen using the CDT-DB database (http://www.cdtdb.neuroinf.jp/CDT/Top.jsp) (S1 Table). Furthermore, to pinpoint genomic regions of interest for DNA methylation analysis, we capitalized on a previous genome-wide study which outlined several specific DNA coordinates that demonstrated changes in cerebellar 5hmC between P7 and P42 (Table 1) [18].

**Table 1:** DNA Methylation Analysis by Restriction Enzyme Digestion Followed by qPCR.

		Genomic Position				% DNA methylation		
Symbol	Chr	GRCm38/mm10 Dec 2011	NCBI37/mm9 July 2007	Szulwach et al., 2011*	Gene Region	P7	P29	p value
*Cacna1g*	11	94475570–94475919	94336884–94337233	94336856–94337487	Promoter to Intron 1	72%	10%	0.0003
	11	94433798–94434145	94295112–94295459	94294907–94295732	Exon 17	3%	6%	0.18
	11	94428990–94429289	94290304–94290603	94290388–94290643	Intron 21 to Intron 22	9%	78%	0.0018
	11	94425603–94425939	94286917–94287253	94286602–94287380	Exon 25 to Intron 25	50%	83%	0.2
*Grid2*	6	64015530–64015858	63965524–63965852	63965601–63965939	Intron 4	112%	42%	0.0037
*Ppp1r17*	6	56011797–56012089	55961791–55962083	N/A	Promoter	95%	102%	0.7
	6	56026217–56026531	55976211–55976525	N/A	Exon 3 to Exon 4	118%	127%	0.86
*Itpr1*	6	108212871–108213125	108162865–108163119	N/A	Promoter to Exon 1	0.07%	0.11%	0.78
	6	108456741–108456940	108406735–108406934	108406713–108407010	Intron 44	0.62%	0.38%	0.23
	6	108550320–108552689	108501381–108501617	108501267–108501528	Downstream	109%	113%	0.11
*Rgs8*	1	153659694–155506824	155506824–155507042	155506651–155507232	Intron 1	23%	46%	0.0026
	1	153671065–153671346	155518195–155518476	155517949–155518550	Intron 4	100%	84%	0.78
*Syt2*	1	134707086–134707325	136603663–136603902	136603350–136604406	Intron 2	95%	38%	0.045

* Szulwach, K. E., Li, X., Li, Y., Song, C. X., Wu, H., Dai, Q., Irier, H., Upadhyay, A. K., Gearing, M., Levey, A. I. et al. (2011). 5-hmC-mediated epigenetic dynamics during postnatal neurodevelopment and aging. Nature neuroscience 14, 1607–1616.

**Fig 7:**
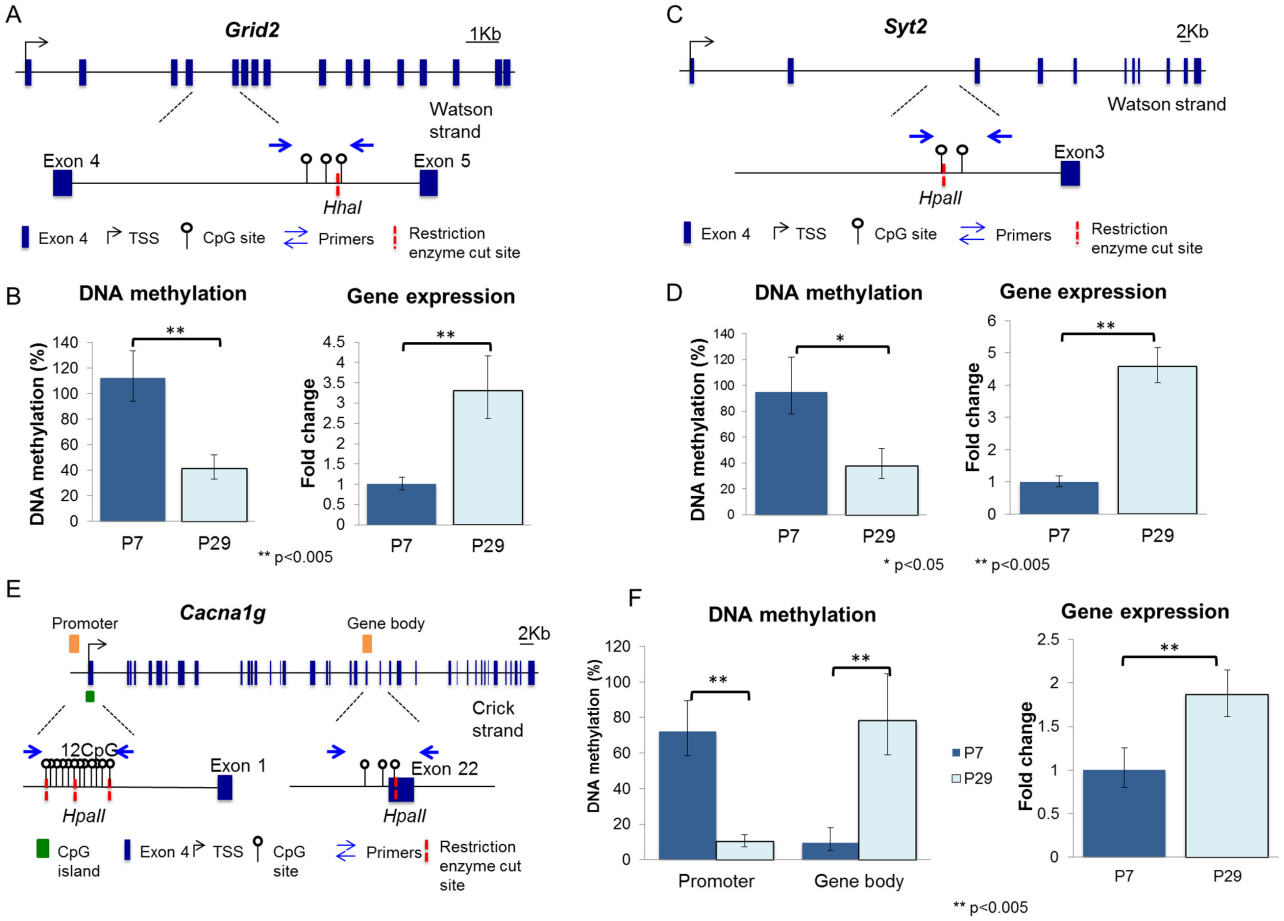
Quantitative detection of DNA methylation in the postnatal cerebellum. (**A**) *Diagram of Grid2 gene structure*. Transcription start site (TSS): black bent arrow; exons: black boxes. *Grid2* is transcribed from the Watson strand. A region in the intron 4 was analyzed. The lower half of the panel is a magnification of the target region. Three CpG dinucleotides and one *HhaI* cleavage site are located in the target region (Chr6:64,015,530–64,015,858). Displaying primer (blue, straight arrow), CpG dinucleotides (white “lollipops”), and restriction enzyme cleavage sites (red, dashed lines). (**B**) *Analysis of Grid2*: This figure represents DNA methylation changes between P7 and P29 following digestion with *HhaI*. The P7 cerebellum is highly methylated, while the P29 cerebellum shows about a 60% reduction in DNA methylation. P-value = 0.0037. *Gene expression of Grid2*: Quantitative RT-PCR of P7 and P29. *Grid2* is expressed 3.3 fold higher in P29 than in P7 cerebellum. P-value = 0.0006. (**C**) *Analysis of Syt2*. We amplified the following genomic region for *Syt2*: Chr1: 136603663+136603902. (**D**) This figure represents DNA methylation changes between P7 and P29 following digestion with *Hpa*II. The P7 cerebellum is almost completely methylated, while P29 cerebellum shows about a 60% reduction in DNA methylation. P-value = 0.045. From P7 to P29, *Syt2* mRNA expression is increased 4.6 –fold. (**E**) *Analysis of Cacna1g*. We amplified the following genomic region for *Cacn1g*: Chr11: 94336884–94337233. (**F**) This figure represents DNA methylation changes between P7 and P29 following digestion with *Hpa* II. The P7 cerebellum is ~ 72% methylated, while P29 cerebellum shows only 10% methylation. P-value = 0.0003. Gene body methylation analysis reveals a reciprocal relationship across ages, where P7 methylation begins around 10% and spikes to about 80% by P29. Overall gene expression was increased 2-fold across this time.

We used methylation-sensitive restriction enzyme digestion followed by quantitative PCR to analyze DNA methylation (5mC and 5hmC) at sites predicted to demonstrate alterations in 5hmC during cerebellar maturation. We found that DNA methylation at some loci increased during differentiation, while other loci demonstrated a loss of methylation. For example *Grid2* (a PC-expressed gene) and *Syt2*, which is detected at the synapse, underwent de-methylation within the gene body by P29. The gene body of *Grid2* showed 100% methylation at P7; this dropped to 42% by P29 (p = 0.0037; Fig 7B). Similarly, *Syt2* demonstrated a marked decrease in DNA methylation within the gene body (95% to 38%; p = 0.045) between P7 and P29 (Fig 7D). However, not all loci underwent de-methylation. We examined DNA methylation at four independent sites in *Cacna1g*, a gene encoding a low voltage calcium channel that is widely detected in neurons. The promoter showed a marked decrease in DNA methylation (72% at P7 to 10% at P29; p = 0.0003), while one site in the gene body shows a marked increase in methylation (9% to 78%; p = 0.0018) between the two stages (Fig 7F). Although two other sites within the gene body of *Cacna1g* showed an increase in DNA methylation at P29, they were not statistically significant. The G-protein signaling regulator gene *Rgs8*, did show a 23% increase in gene body DNA methylation between P7 (23%) and P29 (46%) (p = 0.0026). Not all examined loci exhibited a developmental DNA methylation reprogramming. For example, the promoter and the gene body of *Ppp1r17* (a regulatory subunit of protein phosphatase 1) were completely methylated through P7 and P29, while the promoter and gene body of *Itpr2* (an inositol triphosphate receptor gene) were virtually devoid of methylation at these two stages.

These data demonstrate that de-methylation does occur throughout various genomic regions implicated in cerebellar development. Some genes show region-specific changes, with profound de-methylation of the promoter and increased DNA methylation in the gene body, while others remain methylated or hypomethylated throughout development. Notably, these genes were analyzed on whole cerebellum, of which PC DNA makes up a smaller fraction. Consequently, analysis of genes which are not characteristically limited to PCs, may be likely underestimated compared to the observed immunocytochemical response, as DNA methylation may either not change, or shift in the opposite direction on non-PC cells.

To understand the functional consequences of alterations in DNA methylation during the CDR, we further analyzed mRNA expression of *Grid2*, *Cacna1g*, *Syt2* and *Rgs8* at P7 and P29. All genes were upregulated by P29 with fold changes of 3.3 (p = 0.0006), 1.9 (p = 0.0037), 4.6 (p = 0.0001) and 10.6 (p = 0.0001), respectively. *Itpr1* and *Ppp1r17* were also found to be upregulated by 3.5 and 4.9 fold, respectively, by P42 in a previous study [18]. DNA methylation in the gene body has been increasingly associated with active transcriptional states [32–34]. While the DNA methylation and gene expression profiles of *Cacna1g* fit this observation, *Grid2* and *Syt2* actually showed a significant decrease in gene body DNA methylation and increase in mRNA expression (Fig 7). Because intragenic distribution and DNA methylation marker (5mC vs. 5hmC) appear to be determinants of transcriptional outcomes [31], reliable methods capable of teasing out the contributions of 5mC and 5hmC methylation will become a necessity in future research.

### The Contrast of Granule and Interneurons

Granule cells, the most abundant neurons in the cerebellum (and the entire brain), also adopt a temporspatially independent DNA methylation program during cerebellar development, expressing a reprogramming that is different in both scale and timing compared to PCs. Hypo-methylated at their proliferating progenitor stage, the granule cells acquire 5mC methylation prenatally, when restricted differentiation occurs at E15, and continues to increase when these cells migrate to the cerebellar primodium at E17 (data not shown). At P7, at the cerebellar superfice, those immature and still-dividing granule precursors residing at the outer limits of the external granular layer (EGL^o^) were largely un-methylated. As the granule precursors exited the cell cycle and descended into the inner EGL (EGL^i^) (also distinguishable by their elongated somata), they underwent *de novo* methylation (5mC), expressing both 5mC and 5hmC by the time they reached the EGL^i^ (Fig 1A and 1D, blue crossed arrows). This observation suggests that asymmetrical division within the progenitor populations, and consequent cell fate specification may be determined through the differential acquisition of various methylation marks. As the granule cells migrate inward from the EGL^i^ toward their target position at the Inner Granule Layer (IGL), 5mC and 5hmC levels further increase in accordance with their maturation stage, peaking as they settle at their final destination (IGL) in contrast to PCs, most granule cells in the IGL maintain the acquired 5mC and 5hmC marks up to P21 (Fig 1, blue crossed arrows).

LC-MD was also used to verify the DNA methylation program of the cerebellar granule cells. Proliferating granule cells of the EGL were compared with post-mitotic granule cells which had completed their radial migration into the IGL by P7. Between the period of cell-cycle arrest and radial migration to the IGL, granule cells of the cerebellum nearly doubled their 5mC expression (Fig 3C, p<0.02). Further, even after granule cells reach the IGL, they gradually continued to acquire 5mC from P7 to P29 (Fig 3D, p<0.007), a time-frame overlapping with the development of granule cell parallel fiber synapse formation (P14-P28) (Paul et al 2012).

Granule and Purkinje cells are not the only cells that demonstrate a temporospatial change in DNA methylation; stellate cells of the Molecular Layer (remnant of the early postnatal EGL), Golgi neurons in the IGL, and basket cells (Fig 1, purple dots) in the PCL, also adopt a DNA methylation program that is spatially and temporally unique to their developmental course. In contrast to the PCs, which undergo the CDR as previously discussed, the non-projection neurons (granule, stellate, basket and Golgi) of the cerebellum maintained their cellular 5mC and 5hmC DNA methylation profiles upon acquisition well into adulthood. Though similar to the granule cell DNA methylation program, the time and space within which interneurons acquire 5mC and 5hmC is unique to their cellular development. Granule cells, as mentioned, acquire 5mC and 5hmC during the first postnatal week, at which time they exit the cell cycle and migrate radially into the target IGL. Stellate and basket interneurons, on the other hand, acquire DNA methylation as they complete their migratory paths around the second postnatal week [35] and retain their signal well after their maturation has been completed (approximately the fourth and fifth postnatal weeks). The distinct programs observed between the two categories of neurons in the cerebellum indicate that there may be a structural and functional complexity to PCs that requires an additional cycle of epigenetic reprogramming to accommodate the well-characterized transcriptional demands of its cellular specialization.

### Downstream DNA De-methylation

**Fig 8:**
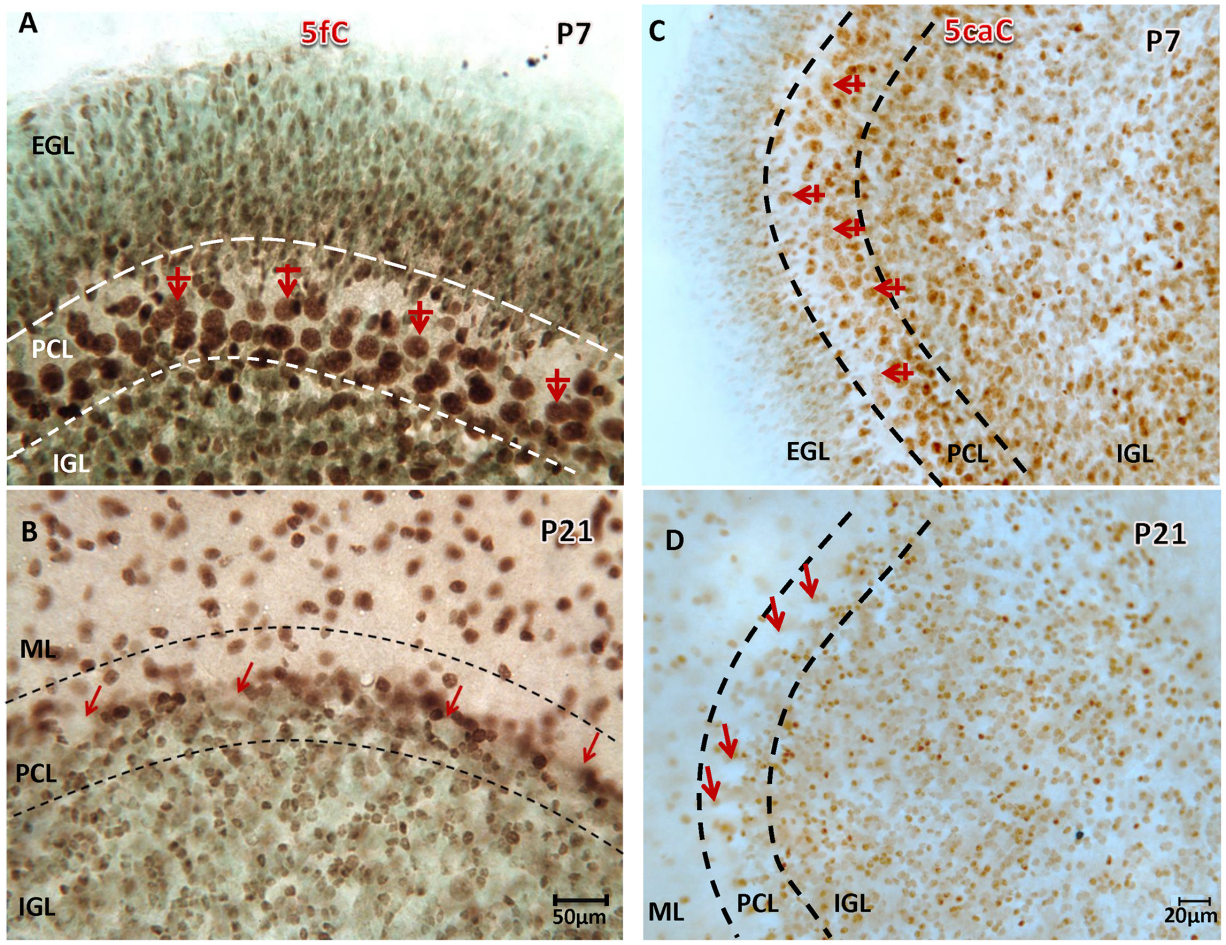
Postnatal loss of 5hmC is synchronized with loss of downstream 5fC and 5caC in PCs. 5fC and 5caC are downstream metabolites of 5hmC (catalyzed by Tet enzymes) and prevail throughout cerebellar neurons including PCs (red, crossed arrow) and post-mitotic granule cells at P7 (**A,C**). As 5hmC is de-methylated at P21 (Fig 1), 5fC and 5caC are also greatly reduced (**B, D**, red arrows). On the other hand, as PCs undergo de-methylation of the 5fC and 5caC derivatives, surrounding basket cells acquire immunoreactivity. IGL: internal granule cell layer, PCL: Purkinje cell layer, ML: molecular layer, Nissl counterstaining: methyl green. Scale bar: **A-B** = 50μm, **C-D** = 20μm.

While the functional implications and mechanistic underpinnings of replication-independent DNA de-methylation remain debated [36], several lines of evidence have demonstrated a methylation-antagonizing role of the Tet enzymes and their downstream DNA methylation derivatives 5fC and 5caC [37, 38]. Here we found that immunostaining of 5fC and 5caC, appearing early at E8 and E10 (not shown), prevailed in the postnatal stage. The distributions of 5fC and 5caC were similar to and highly coordinated with the distribution of 5mC (Fig 8). The 5caC and 5fC were detected in abundance in PCs at P7, but were no longer detectable at P21-P28, in line with 5mC and 5hmC loss.

## Discussion

### Purkinje Cells Showcase the Methylation and De-methylation Program

Although we have previously observed the wax and wane of DNA methylation during neuroepithelial cell differentiation during neural tube [20, 21] and hippocampus development [22], their turn-over occurs on a relatively moderate scale. Purkinje Cells are a larger type of neuron and are characterized by the largest dendritic arborization known to occur in the mammalian brain. The morphological transition occurring from the primordial cell division to full size mature PC specification is an extreme progression and requires large scale gene transcription turnover. This perhaps provides an exceptional opportunity to document the DNA methylation program (turnover) which may also occur in other, more subtle neuronal transformations at less discernible levels. The dramatic CDR of the cerebellar PCs was consistent across several DNA methylation markers as well as in single-cell population gDNA examination. The distinct size of the PCs (~30 μm) during de-methylation and their reliable position in the PCL allowed for the clear distinction of the population during immunodetection and laser microdissection. To rule out PC loss of immunoreactivity or incomplete immunopenatrance during de-methylation, the ubiquitous PC marker calbindin was examined and consistently detectable throughout PC development. Finally, complimentary epigenetic reprogramming was immunocytologically detectable in the PCL-surrounding basket interneurons, at every section depth. Collectively, our results indicate a complete and highly scheduled CDR intrinsic to each class of cerebellar cell population illustrating a role for the DNA methylation program during neural differentiation.

### Evidence and Diversity of Cell-wide Neuronal De-methylation

**Fig 9:**
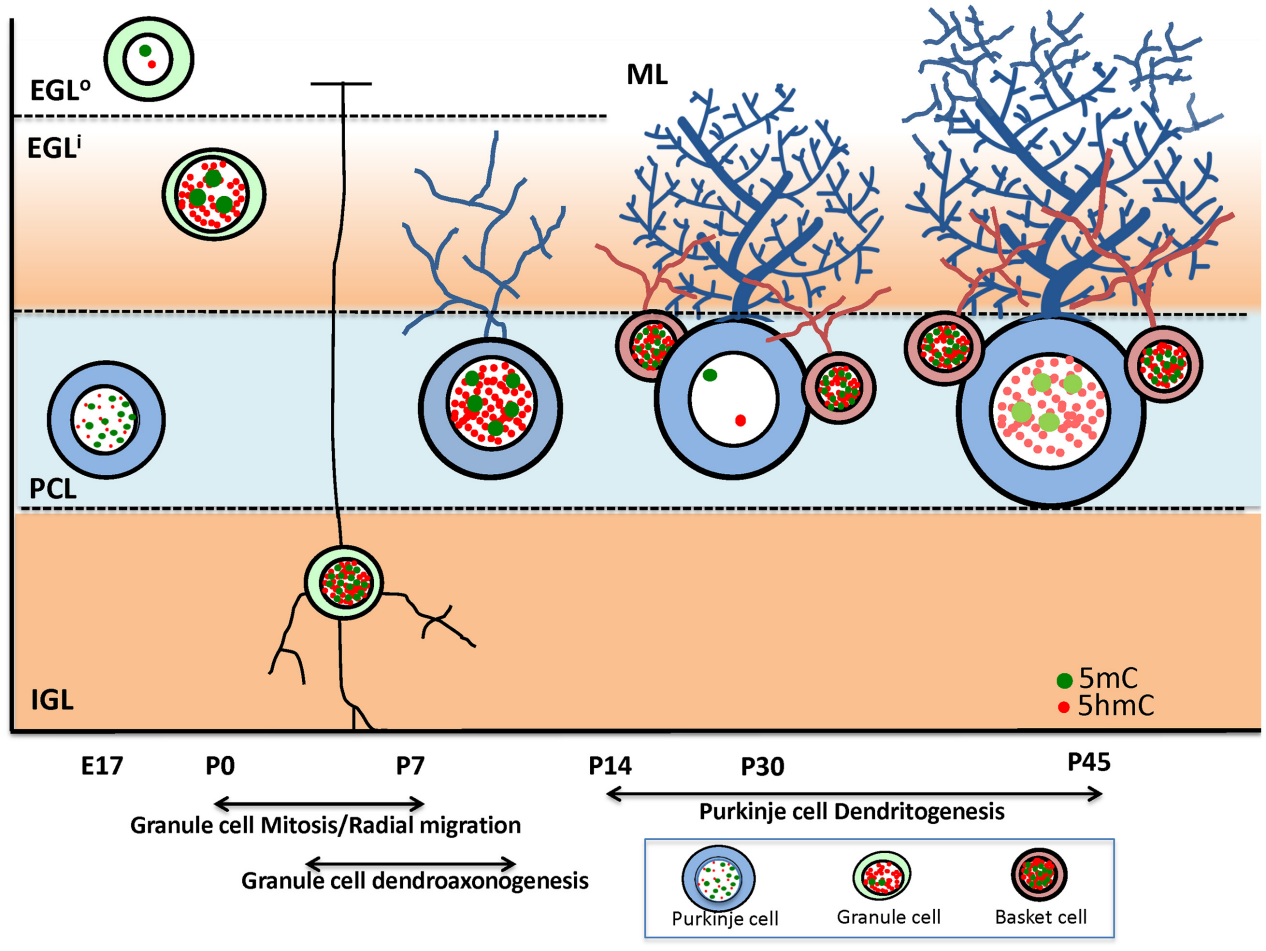
Independent DNA methylation program of Purkinje, granule and basket cells during development. This scheme illustrates the cell-specific epigenetic distribution of the DNA methylation marks 5mC (green dots) and 5hmC (red dots) in the nuclei of neurons during development of the cerebellum. As cerebellar granule cells (light green) occupy the outer external granule layer (EGL^o^), they are still mitotic and devoid of 5mC and 5hmC. Immediately after completing mitosis, these granule cells exit the cell cycle and begin radial migration through the inner EGL, PCL, and finally into the internal granule layer (IGL). As soon as granule cells break through the EGL^o^, they strongly acquire 5mC and 5hmC, though these two methylation marks are independently distributed in the nuclei of granule cells. From P7 forward, mature granule cells of the IGL maintain their methylation, though these become homogenously distributed in contrast to pre-migration distribution. Independently, Purkinje cells (PC) of the cerebellum exhibit a unique epigenetic program. Post-mitotic Purkinje cells generated in the dorsal rhomboid lip at E14 and arrived at the Purkinje cell layer (PCL) at E17 and already express 5mC quite prominently (and to a lesser extent 5hmC). As the PC grow in size, it becomes clear that 5mC are distributed in a granular fashion in heterochromatin, while 5hmC are distributed as fine particles in euchromatin. Remarkably, just prior to PC’s undergoing characteristic dendritogenesis and synaptogenesis (P14-30), a dramatic loss of 5mC and 5hmC occurs in their nuclei. As Purkinje cells settle into synaptic maturity, 5mC and 5hmC reappear in the nuclei though diminished from peak levels observed at P7. In contrast, the basket interneurons closely associated with PCs appear to have acquired a rich expression of 5mC and 5hmC while PCs undergo de-methylation and re-methylation.

It has been long and widely speculated that cells and tissues have their specific methylation signatures to achieve their diversity and specialization. Some comparative studies have found that gene-specific methylation can vary within neuronal cell types [39]. Despite this, little has been elucidated about cell-specific differential methylation. We understand during development how dramatic change of germ line to somatic cell or pre- to post-mitotic cells is paralleled with transcriptional and morphological change, yet the prevalence of a cell-specific DNA methylation evolution as a viable mechanism is largely unknown. Here, we witnessed that cell-wide methylation changes occurred in parallel with the large scale of transcriptional and morphological changes of the cerebellar PCs (Fig 9). The PCs, acquiring a first wave of 5mC and 5hmC methylation as they transition perinatally into their target PCL, subsequently require completely different machinery to accommodate the demand for arborization and functional connectivity. We found at this junction (2^nd^ to 4^th^ week postnatal weeks), a cell-wide de-methylation is indicated by cell-specific immunocytochemistry, laser capture-global molecular DNA methylation assay, and PC-gene-specific methylation assay. Upon completion of the active arborization and synaptogenesis, re-methylation occurred. These observations are supported by the parallel turn-over of de-methylation markers, methylation enzymes, and by the downstream DNA methylation binding proteins.

In contrast the granule cells, which undergo their own cytological transition from pre- to post-mitotic state and radial migration from the EGL^o^ to EGL^i^ (and further from EGL to IGL), express an independent methylation program, as indicated by cell-specific immunocytochemistry and laser capture-global molecular methylation assay. These together illustrated a clear picture of a cell-specific DNA methylation program working parallel to cell-specific, temporospatial differentiation (Fig 9).

### Significance of the Cell-wide De-methylation

Since cell-wide de-methylation reprogramming is evolutionally well-preserved, it is likely to serve an indispensable biological function. Although it occurs now in broader cellular profiles (preimplantation embryos, oocytes, spermatocytes, and maturing neurons) at diverse stages (from single cell embryos to postnatal brain development), they share a fundamental commonality––initiating a developmental transition which requires a tremendous transcriptional overhaul. Cell wide methylation reprogramming could likely serve as a mechanism to allow or facilitate this transition. Gene expression studies from other groups indeed indicate that changes in gene expression profiles accompany differential methylation profiles [40, 41]. In the cerebellum, transcript levels of cell cycle genes were greatly reduced when 5mC reached its highest level and consolidated in the heterochromatin at P7, while large number of genes involved in the neuroskeleton and synaptogenesis pathways was highly upregulated from P14 to P42 [18] when de-methylation occurred. Interestingly, overall transcript levels of Purkinje specific genes increased dramatically between P7 and P42 [18, 42]. These significant profile changes in PCs occur in synchrony with the robust cellular growth and neuronal specialization known to occur in the first few weeks of postnatal cerebellum development (Fig 9). Particularly, the PCs undergo one of the most robust dendritic expansions known in the brain. In a rat brain developmental analysis, researchers calculated that between the first and second postnatal week, dendritic area grows to as much as 10,000 um^2^ [28]. This dendritic expansion and accompanying synaptogenesis and axonal growth occurs during the time frame fitted to the cell-wide de-methylation program we report here. It is plausible then that de-methylation serves as a general gene-activating mechanism across a wide spectrum of genes that mediate neuronal growth, synaptogenesis, and receptor formation.

After the major observed de-methylation, a *de novo* re-methylation of 5mC and 5hmC begins topographically from the surface (where cells mature earlier) to the inner folds of the cerebellar lobule (where cells mature later). Coincidentally, this is the time around which dendritosynaptic growth tapers off and by P45, a majority of the PCs in the cerebellum are re-methylated with both 5mC and 5hmC (Fig 1). The epigenetic correlate MECP2 also returned at this point in this orchestration (not shown). If de-methylation plays a functional role in the activation of genes of synaptogenesis, subsequent re-methylation may restore the repressive state of those developmental genes. Finally, it should be noted that the cell-wide we have observed across multiple cell types is not absolute. This leaves many methylation marks intact, probably to keep undesirable, spurious transcription in check (e.g. X-inactivation, transposons) or to direct allele-specific repression (e.g. imprinting genes).

PCs demonstrate the greatest CDR among all neurons we have analyzed to-date [20, 22]. They are also one of the one of the largest neurons (25–40μm in diameter) in the brain and have the most extensive dendritic profile including synaptic and receptor molecules. Sensibly, a large scale epigenetic reprogramming event such as the one we have characterized may be needed to assure the morphological and molecular changes so distinctively dramatic of PCs. On the other hand, various degrees of smaller scale de-methylation may also occur in a wide-range of cells where and when transcription is required for more subtle cellular transitions during development or for plasticity in coping with environmental changes throughout adulthood. The latter have been regionally noted in activity-dependent assessments of DNA methylation status [14–16].

### Potential Mechanism of Cell-wide De-methylation

Despite the recent successes in identifying that the TET enzymes oxidize 5mC to 5hmC, the mechanisms and processes underlying DNA de-methylation remain elusive. At the pronuclear stage, initial de-methylation of the paternal genome is an active process mediated by TET3 [43, 44]. The maternal germline initially escapes this de-methylation because the presence of DPPA3 prohibits TET3 binding [45, 46]. During early pre-implantation development and germ line maturation both passive and active DNA de-methylation occurs. Because PCs at P7 to P21 are post-mitotic, we expect that the decreases in 5mC and 5hmC methylation occur through active, TET-mediated de-methylation events. In support of this, we found that initiation of TET enzyme expression is cell specific and highly substrate dependent in a temporal manner. TET1/TET2 appear hours to days after the presence of 5mC in the developing brain. However, the appearance of the TET enzymes coincides with the detection of 5hmC during pre- and postnatal differentiation of the cerebellum and forebrain (not shown). This is in agreement with Song and colleagues [14] who found that TET1 mediates environmentally-induced oxidation-deamination of 5mC to 5hmC *in vivo*. Therefore, the short delay in the appearance of 5hmC behind 5mC is apparently due to the timely arrival of TET1/TET2.

The turnover of DNMT1 and TET1 in PCs also supports the de-methylation events that we outline in this study. At P7, both TET1/TET2 were highly expressed in a concordant manner with 5mC and 5hmC in PCs. By P21, both enzymes severely diminished in synchrony with the reduction in 5mC and 5hmC (Fig 5). In contrast, in the granular, stellate, basket and Golgi neurons, TET1 and TET2 remained constant, and occurred in conjunction with the presence of 5mC and 5hmC (Fig 5). Thus, TET enzymes closely mediate the progression of the DNA de-methylation program in a cell-specific manner, and we advocate that the TET enzymes perform a critical task in the progression of individual neural development.

Recent studies demonstrated that the TET family of proteins are capable not only of converting 5mC to 5hmC [47, 48], but also can further oxidize 5hmC to 5-formylcytosine (5fC) and 5-carboxylcytosine (5caC) [49]. 5caC can be excised by thymine-DNA glycosylase (TDG)–an enzyme which mediates base excision repair yielding unmethylated cytosine residues [37]. Although functionally unclear, the 5fC and 5caC exist quite early during neural tube formation and continuously into the postnatal stage. These findings indicate that either (a) there is a yet-to-be defined pathway for the formation of 5caC and 5fC that is independent of 5mC de-methylation, or (b) there is a constant turnover of DNA methylation throughout development in these cells. Turnover of the 5caC and 5fC also supports the observed de-methylation in PCs. When 5mC and 5hmC were erased at over the period of the 2^nd^ to 4^th^ week, the 5caC and 5fC were also reduced. In contrast, all DNA methylation marks (5mC, 5hmC, 5caC and 5fC) were present in granular neurons and all interneurons in the cerebellum at P21. We believe that turnover of 5caC and 5fC is occurring during neonatal development, since 5caC and 5fC are prominent in developing cells and are unfailing partners of 5mC/5hmC spatiotemporally, they could play a yet-to-be identified function during development.

## Conclusions

In conclusion, our findings forward the concept that large-scale cell-wide DNA methylation and de-methylation-re-methylation events comprise a normal program during growth and specialization of post-mitotic neurons, indicating that this type of event is not restricted to preimplantation embryos and the germ line. The fact that this CDR occurs in specific neuronal cell types clearly impacts high-throughput analysis and argues that cell type separation and single cell analyses should be considered in future studies. In large neurons, such as Purkinje cells, with enormous post-mitotic growth (e.g. huge axodendritic growth, synaptogenesis, and channel / receptor formation), wide cellular de-methylation occurs. This current report, along with previous neural tube studies [20, 21], indicate that DNA methylation programming occurs in a spatiotemporal and cell specific manner coincident with neuronal differentiation. Given the function of DNA methylation in chromatin remodeling, if the intricate and accurate DNA de-methylation and re-methylation events are required for brain maturation through adolescence, then alterations in DNA methylation by environmental factors could profoundly influence development.

## Supporting Information

S1 Table Developmental Gene Regulation of Purkinje Cells and Cerebellar Synaptic Targets. Developmental gene expression changes in Purkinje cell characteristic and synaptic genes. **A**. Table of genes predominantly expressed in Purkinje cells of the cerebellum and their expression levels at comparable stages of postnatal development. **B**. Table of genes playing prominent roles in synapse formation in the brain and their expression levels at comparable stages of postnatal development. Data compiled from CDT-DB database (http://www.cdtdb.neuroinf.jp/CDT/Top.jsp)* and Szulwach et al 2011^**†**^.
(DOCX)
