# Supplementary material for: Cell-Wide DNA De-Methylation and Re-Methylation of Purkinje Neurons in the Developing Cerebellum
Source: PLoS One. 2016 Sep 1;11(9):e0162063. doi: 10.1371/journal.pone.0162063 (PMC5008790; doi:10.1371/journal.pone.0162063)
Supplement: S1 Table — Developmental gene expression changes in Purkinje cell characteristic and synaptic genes. A. Table of genes predominantly expressed in Purkinje cells of the cerebellum and their expression levels at comparable stages of postnatal development. B. Table of genes playing prominent roles in synapse formation in the brain and their expression levels at comparable stages of postnatal development. Data compiled from CDT-DB database (http://www.cdtdb.neuroinf.jp/CDT/Top.jsp)* and Szulwach et al 2011†. (DOCX) [file pone.0162063.s001.docx]

**SUPPLEMENTARY MATERIAL**

**S1 Table. Developmental Gene Regulation of Purkinje Cells and Cerebellar Synaptic Targets**

| **A. Purkinje Specific Genes** | | | | | **Gene expression (fold change)** | | |
| --- | --- | --- | --- | --- | --- | --- | --- |
| **Symbol** | **Description** | | | | **P21 vs P7** | **P42 vs P7** | |
| **Atp2a3** | **ATPase, Ca++ transporting, ubiquitous** | | | | **4.46** | **3.82** |  |
| **Baiap2** | **brain-specific angiogenesis inhibitor 1-associated protein 2** | | | | **1.73** | **1.48** |  |
| **Cacna1g** | **calcium channel, voltage-dependent, T type, alpha 1G subunit** | | | | **1.93** | **-1.03** |  |
| **Calb1** | **calbindin 1** | | | | **1.41** | **2.29** |  |
| **Car8** | **carbonic anhydrase VIII** | | | | **2.11** | **2.73** |  |
| **Gsbs (Pp1r17)** | **Protein phosphatase 1, regulatory subunit 17** | | | | **11.9** | **7.76** |  |
| **Gria1** | **glutamate receptor, ionotropic, AMPA1 a(α1)** | | | | **2.21** | **1.43** |  |
| **Grid2** | **glutamate receptor, ionotropic, δ2** | | | | **2.34** | **1.66** |  |
| **Homer3** | **homer homolog 3 (Drosophila)** | | | | **2.94** | **2.12** |  |
| **Itpr1** | **inositol 1,4,5-triphosphate receptor 1** | | | | **3.6** | **4.88** |  |
| **Pcp2** | **Purkinje cell protein 2 (L7)** | | | | **1.99** | **3.46** |  |
| **Pcp4** | **Purkinje cell protein 4** | | | | **1.62** | **1.57** |  |
| **Slc1a6** | **solute carrier family 1 (high affinity aspartate/glutamate transporter), member 6** | | | | **1.97** | **1.36** |  |
| **Trpc3** | **transient receptor potential cation channel, subfamily C, member 3** | | | | **4.29** | **1.61** |  |
|  |  | | | |  |  |  |
| **B. Synaptic genes:** | |  | **Gene expression (fold change)** | | | | |
| **Symbol** | **Description** | | |  | **P21 vs P7** | **P42 vs P7** |  |
| **Fgf14** | **fibroblast growth factor 14** | | | | **3.89** | **1.59** |  |
| **Grip1** | **Glutamate receptor-interacting protein 1** | | | | **-5.8** | **-2.72** |  |
| **Nlgn1** | **Neuroligin-1** | | | | **1.17** | **-1.51** |  |
| **Nrxn2** | **neurexin II** | | | | **1.68** | **1.02** |  |
| **Plxnb2** | **cell adhesion and recognition-related gene; neurite growth and synapse formation-related gene** | | | | **-2.19** | **-1.21** |  |
| **Rgs8** | **regulator of G-protein signaling 8** | | | | **7.09** | **2.82** |  |
| **Rims1** | **Regulating synaptic membrane exocytosis 1** | | | | **3.29** | **1.88** |  |
| **Syt2** | **SNARE synaptotagmin II** | | | | **4.77** | **2.3** |  |
|  |  | | | | **(CDT-DB database;** | **(Szulwach at el 2011)** |  |
|  |  | | | | http://www.cdtdb.neuroinf.jp/CDT/Top.jsp ) |  |  |

Developmental gene expression changes in Purkinje cell characteristic and synaptic genes. **A**. Table of genes predominantly expressed in Purkinje cells of the cerebellum and their expression levels at comparable stages of postnatal development. **B**. Table of genes playing prominent roles in synapse formation in the brain and their expression levels at comparable stages of postnatal development. Data compiled from CDT-DB database (<http://www.cdtdb.neuroinf.jp/CDT/Top.jsp>)* and Szulwach et al 2011**^†^.**
